# Supplementary material for: Evaluation of Bi-Lateral Co-Infections and Antibiotic Resistance Rates among COVID-19 Patients
Source: Antibiotics (Basel). 2022 Feb 19;11(2):276. doi: 10.3390/antibiotics11020276 (PMC8868529; doi:10.3390/antibiotics11020276)
Supplement: Supplementary file 1 [file antibiotics-11-00276-s001.zip › antibiotics-1586757-supplementary.pdf]

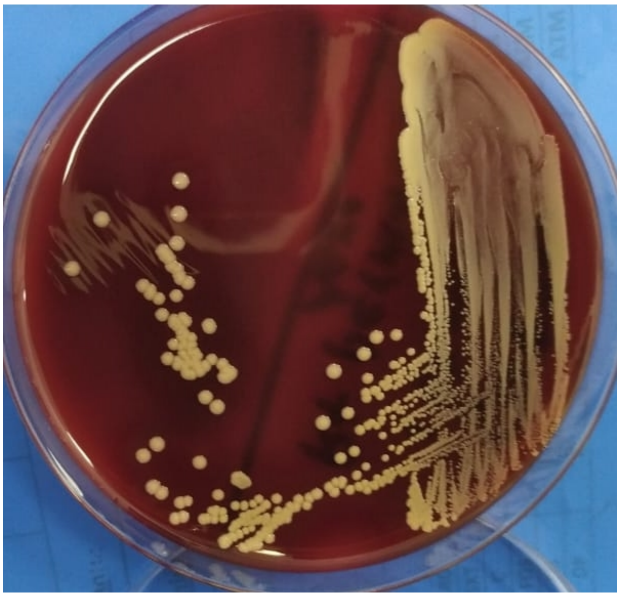

**Figure S1.** Growth of catalase and coagulase positive (*Staphylococcus aureus*) bacteria on blood agar plate.

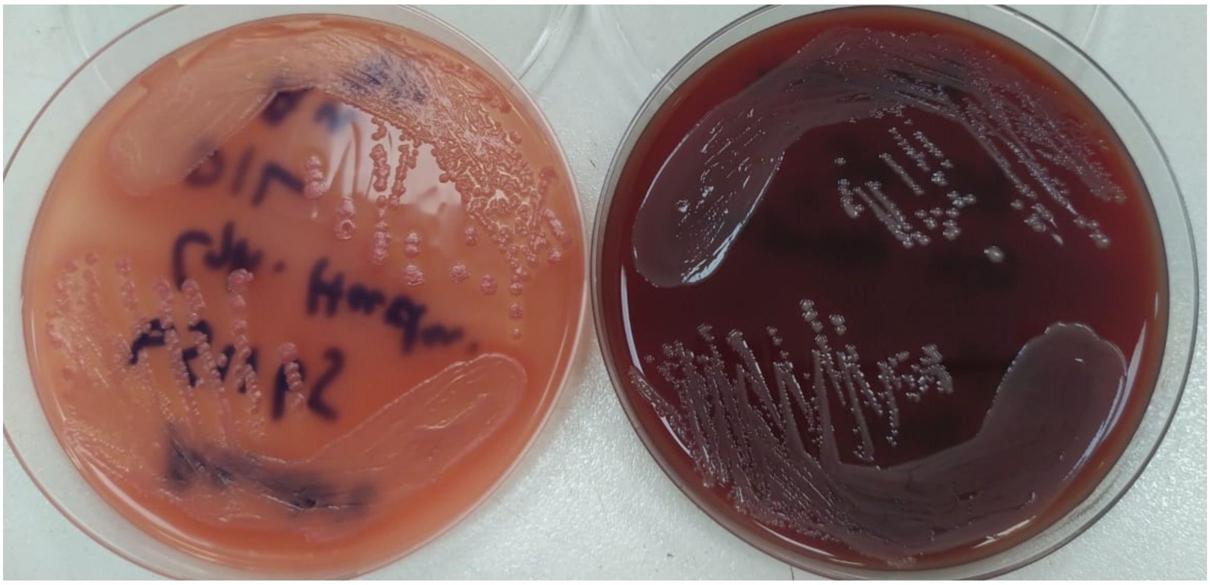

**Figure S2.** Growth of gram-negative bacteria (Lactose fermenter) on blood and MacConkey agar plates after 18 hours of incubation period at 37°C.

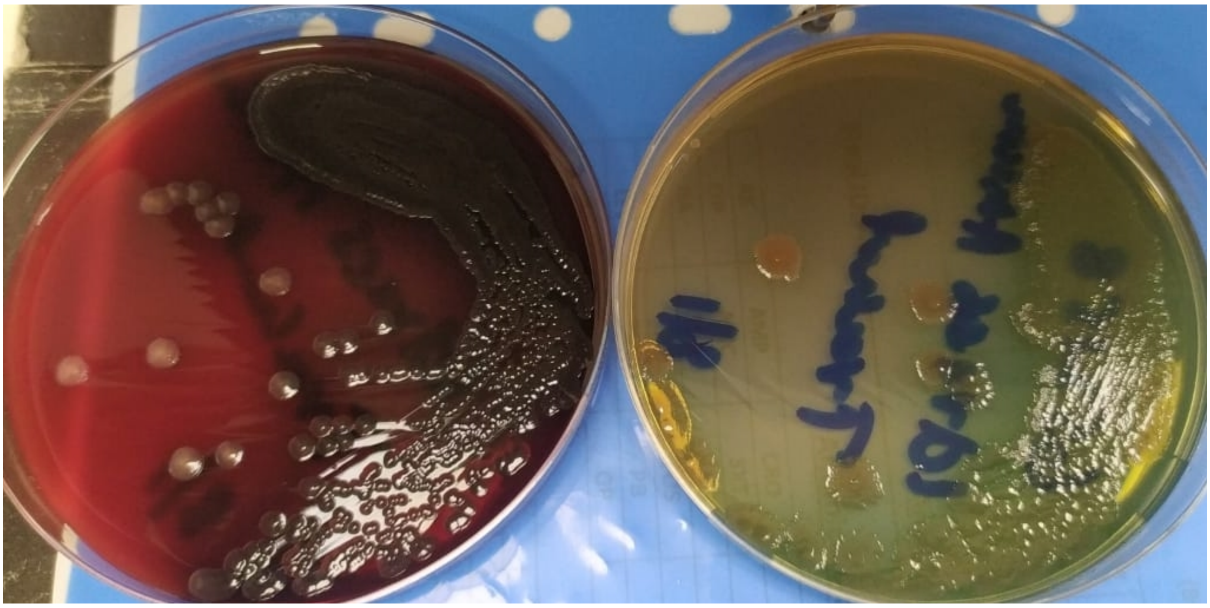

**Figure S3.** Growth of oxidase positive bacteria (probably *Pseudomonas aeruginosa*) on blood and CLED agar plates.

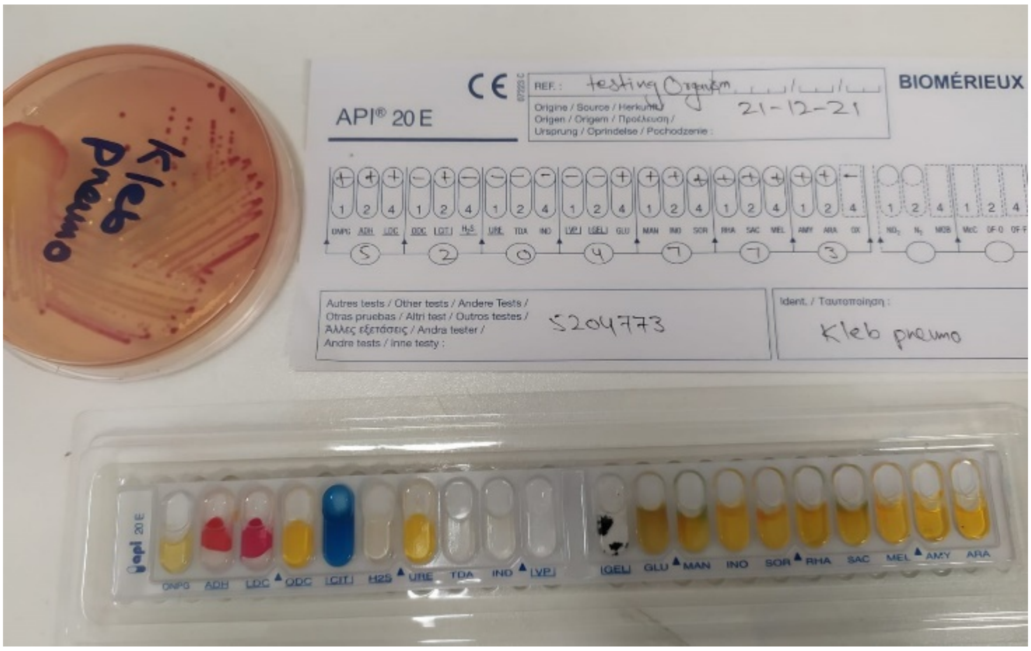


**Figure S4.** In given picture API 20E strip has been shown, on reading page all results of wells are given. On the bases of results, a unique number was produced which was matched with data bank available on BIOMEUREX website. We had used different types of APIs for different organisms, like API NE for non-Enterobacteriaceae and many more.

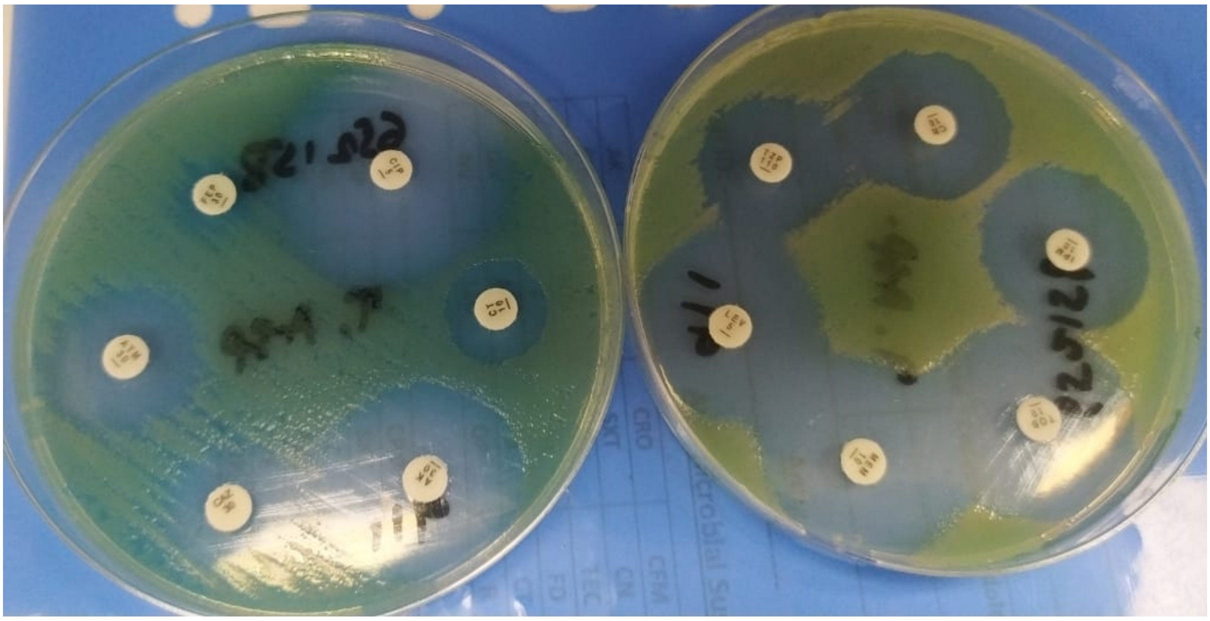

**Figure S5.** Sensitivity pattern of *Pseudomonas aeruginosa* on MH agar plates.

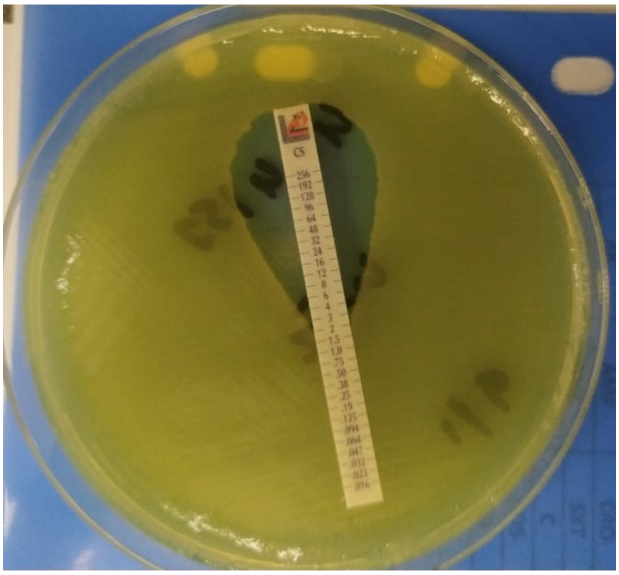

**Figure S6.** MIC of Colistin on MH agar plate.

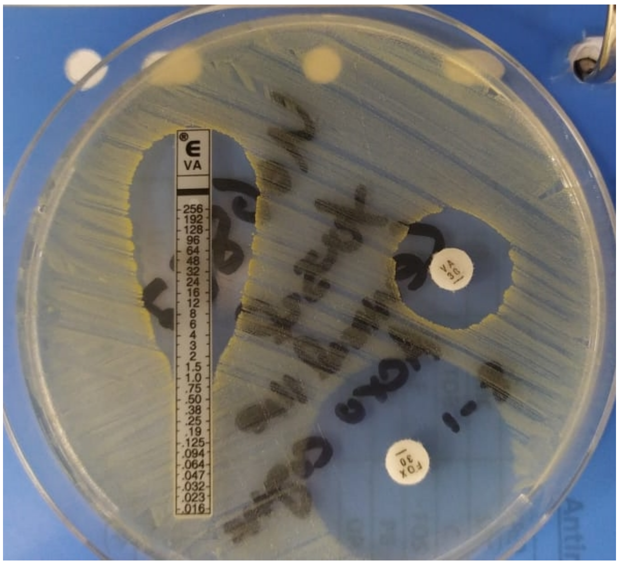


**Figure S7.** MIC of Vancomycin on MH agar plate with Sensitivity pattern of Vancomycin (Sensitive) and Cefoxitin (Sensitive) discs.

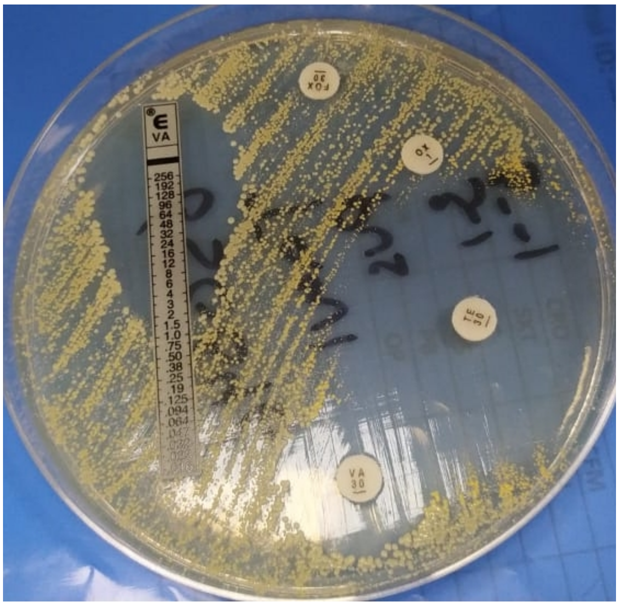


**Figure S8.** MIC of Vancomycin on MH agar plate with Sensitivity pattern of Vancomycin (Sensitive) and Cefoxitin (Resistant), Oxacillin (Resistant), and Tetracycline (Sensitive) discs.
